# Prevalence of and risk factors for hepatitis C virus antibody among people who inject drugs in Cambodia: a national biological and behavioral survey

**DOI:** 10.1186/s12954-019-0299-1

**Published:** 2019-04-29

**Authors:** Siyan Yi, Phalkun Mun, Pheak Chhoun, Navy Chann, Sovannary Tuot, Gitau Mburu

**Affiliations:** 1 Saw Swee Hock School of Public Health, National University of Singapore and National University Health System, Tahir Foundation Building, 12 Science Drive 2, #10-01, Singapore, 117549 Singapore; 2 KHANA Center for Population Health Research, Phnom Penh, Cambodia; 3 Center for Global Health Research, Touro University California, Vallejo, USA; 4 National Center for HIV/AIDS, Dermatology and STD, Phnom Penh, Cambodia; 5 Division of Health Research, Lancaster University, Lancaster, United Kingdom

**Keywords:** Co-infection, Harm reduction, Injecting drug use, Key population, Resource-limited setting, Viral hepatitis

## Abstract

**Background:**

Hepatitis C virus (HCV) is a significant global health concern. Despite evidence of the relationship between injecting drug use and HCV, studies on HCV among people who inject drugs in developing countries remain scarce. To address this need, we conducted this study to explore the prevalence of and factors associated with HCV antibody positivity among people who inject drugs in Cambodia.

**Methods:**

Data used for this study were collected as part of the National Integrated Biological and Behavioral Survey among people who use and inject drugs conducted in 2017. We used the respondent-driven sampling method to recruit participants in 12 provinces for face-to-face interviews and HIV and HCV antibody testing. Weighted multivariable logistic regression analysis was conducted to identify risk factors associated with HCV antibody positivity.

**Results:**

This study included 286 people who inject drugs with a mean age of 31.6 (SD = 7.5) years. The prevalence of HCV antibody among participants in this study was 30.4%, of whom 31.0% were co-infected with HIV. After adjustment for other covariates, the odds of HCV antibody positivity was significantly higher among participants who were in the older age group of 25 to 34 (AOR = 1.85, 95% CI = 1.06–7.92) and ≥ 35 (AOR = 2.67, 95% CI = 1.24–5.71), were in Vietnamese ethnic group (AOR = 5.44, 95% CI = 2.25–13.14), were living on the streets (AOR = 3.01, 95% CI = 1.29–704), had been sent to a drug rehabilitation center in the past 12 months (AOR = 2.67, 95% CI = 1.21–5.90), had received methadone maintenance therapy in the past 12 months (AOR = 3.02, 95% CI = 1.32–6.92), and were tested positive for HIV (AOR = 3.80, 95% CI = 1.58–9.12) compared to their respective reference group.

**Conclusion:**

The prevalence of HCV antibody among people who inject drugs in Cambodia is high, particularly in older and more vulnerable subgroups. Tailor-made interventions are required to increase access to culturally sensitive harm reduction interventions to prevent primary HCV infection and reinfection. In addition, there is an opportunity to expand screening, diagnosis, and treatment with new directly acting antiviral agents.

## Background

Hepatitis C is a significant global health problem [1]. The global prevalence of hepatitis C virus (HCV) is estimated at 3%, translating to at least 185 million people living with the infection worldwide [2]. Persistent HCV infection is a leading cause of hepatic cirrhosis, hepatocellular cancer, and liver failure [3]. In 2015, an estimated 495,000 deaths occurred worldwide attributable to HCV [4]. Besides high mortality rates, HCV infection is associated with significant healthcare costs [5].

While the global incidence patterns of HCV have shifted significantly in recent years [2, 6], it has remained a common health problem among people who inject drugs (PWID) [6, 7]. In Cambodia, PWID are a priority population for human immunodeficiency virus (HIV) and harm reduction programming. However, much of the current efforts relate to HIV prevention and treatment among this population [8]. The current focus on prevention and treatment of HIV among PWID is reasonable given the very high HIV prevalence among this group nationally, at 24.8% [9]. In this context, the Cambodian Ministry of Health has set a goal to eliminate new HIV infection by 2025 through the implementation of the Cambodia 3.0 Strategy that focuses on key populations such as PWID, female entertainment workers, men who have sex with men, and transgender women [10]. Consequently, a large part of the existing strategic information related to PWID in Cambodia is concerned with HIV, with relatively limited research having been conducted on HCV epidemiology in this population. While a limited number of studies on HCV epidemiology have been conducted in Cambodia, those studies have mostly focused on the general population [11–13]. Where PWID have been included in these studies, their proportion was small [11].

Injecting drug use is a key driving factor for both HCV and HIV, particularly when injecting is conducted through shared equipment [14]. This situation calls for increased focus on PWID. PWID infected with HCV represent a high-risk group in terms of unsafe injecting practices. Identifying those at high risk for HCV infection could inform education efforts for safer injection among PWID who have not been reached by such services. Such efforts could also contribute to prevention of co-infection with HIV among those already infected with HCV. Early detection of HCV could lead to earlier treatment, given the increasing availability of better-tolerated oral antiviral drugs [15, 16]. Currently, access and treatment to pegylated interferon and ribavirin is very limited in Cambodia [11].

Given the health impacts of HCV infection, the potential for HCV/HIV co-infection, the national goal of better responding to the needs of key and vulnerable populations, and the limited data related to HCV among PWID in resource-limited settings, we conducted a study aiming to determine the prevalence and correlates of HCV antibody positivity in this population in Cambodia so as to inform services, policy, and future research programs.

## Methods

### Study design, sites, and participants

Data used for this study were obtained from the National Biological and Behavioral Survey (IBBS) conducted among people who use and inject drugs from June to December 2017 in the capital city of Phnom Penh and other 11 major provinces. The selection of the study sites was based on a feasibility assessment conducted by the study team prior to the main study. These 12 study sites contained 21 operational districts with high-burden of HIV and large population size of people who use and inject drugs. PWID were defined as people who have injected any types of illicit drugs, defined by the Cambodian Law on Control of Drugs, in the past 12 months [17]. To be included in the study, an individual must (1) be at least 18 years old, (2) have a valid study coupon, (3) have not participated in this survey, (4) meet the definition of PWID, and (5) be able and willing to provide a written informed consent to participate in the study.

### Sample size and sampling procedures

The sample size was calculated for the parent study based on an assumption that HIV prevalence would have decreased by 20% between the national survey in 2012 and the current IBBS and the estimated PWID population size of 1300 [9]. An estimated HIV prevalence of 20% was used, with a margin error of 1.5%, a confidence interval of 95%, a response rate of 90%, and a design effect of 1.5. The minimum required sample size for this study was approximately 280. For the overall IBBS, the sample size was stratified by study sites based on the estimated population size of people who use non-injecting drugs (PWUD), and roughly 15% of the estimated PWUD in each site were recruited. Data collection was conducted in 21 locations (six locations in Phnom Penh and 15 locations in the remaining provinces), determined based on the required sample size of PWUD in each study site. Approximately 25.0% of the estimated 1068 PWID in Phnom Penh in 2016 [18] were recruited, assuming that there would be no PWID in provinces outside the capital city according to HIV programs reports. However, all PWID found in provinces were included in the study.

The respondent-driven sampling (RDS) method was used to recruit study participants, and the Strengthening the Reporting of Observational Studies in Epidemiology for RDS Studies (STROBE-RDS) statement was followed [19]. The RDS was implemented in five stages. First, eligible four seeds who were well connected to other PWID or PWUD in each location were selected with support from non-governmental organizations (NGOs) working in the area. Second, after obtaining a written informed consent, a personal identification number (PIN) was assigned to each seed to be enrolled as a participant. Third, three coupons were given to each seed to refer three additional PWID. For a successful referral, the seed would receive US$2, and the seed was expected to extend to 3–6 “recruitment waves” in each site. Additional seeds would be selected in case the enrolment has been halted because all recruitment chains have “dried up” (i.e., stopped recruiting) or the initial seeds did not recruit participants. Finally, recruited individuals were invited to become seeds and recruit other peers.

### Data collection training

A data collection training was conducted for three days for all data research team members. The contents of the training included informed consent process, interview techniques, confidentiality and privacy, and practices of the questionnaire administration. The teams also reviewed the study protocol and tools, so that all members would be thoroughly familiar with them. A laboratory refresher training was also provided to data collection teams responsible for HCV and HIV testing. Team leaders were also encouraged to perform daily reviews with the team members to review progress and communicate any issues that may occur during the data collection.

### Data collection procedures and tool

#### HCV and HIV test

Rapid serological tests for HCV antibody was performed by a laboratory technician from Médecins Sans Frontières (MSF) in Cambodia with capillary blood using OraQuick© (OraSure Technologies, Inc., Bethlehem). OraQuick© is a World Health Organization (WHO) pre-qualified HCV rapid qualitative immunoassay screening test based on detection of immunoglobulin G HCV antibody [20]. A reactive test establishes that a person had been exposed to HCV resulting in the generation of HCV antibodies. The newly detected HCV antibody positive cases were followed up by MSF for further management.

Rapid HIV testing was performed by a laboratory technician from the provincial health department using SD Bioline HIV/Syphilis Duo test (Standard Diagnostic, Inc., Korea). The SD Bioline HIV/Syphilis Duo test is a WHO pre-qualified solid-phase immunoassay for the qualitative detection of antibodies to all isotypes (IgG, IgM, and IgA) specific to HIV-1/2 and/or *Treponema pallidum* simultaneously [21]. A non HIV-reactive result establishes that an individual is not HIV-infected. A confirmatory test was conducted on site using HIV 1/2 STAT-PAK® Assay (Chembio Diagnostic Systems, Inc., New York) for cases with an HIV reactive result from the SD Bioline HIV/Syphilis Duo test, in keeping with the national HIV testing guidelines. The HIV 1/2 STAT-PAK™ Assay is a rapid immunochromatographic screening test for the detection of HIV 1/2 antibodies [22]. Participants received their results verbally in a post-test counseling session after the interview. New HIV-positive cases were linked to a local NGO working in the area. After completion of the data collection, participants received a gift for their time and transport compensation, costing approximately US$5.

#### Questionnaire development and measures

A structured questionnaire was developed using standardized tools adapted from previous studies among HIV key populations in Cambodia [9, 23–25]. The questionnaire was initially developed in English and then translated into Khmer, the national language of Cambodia. Back-translation was performed to ensure that the “content and spirit” of every original item were maintained. A validated workshop was conducted with representatives of beneficiaries, communities, and key stakeholders working on HIV and harm reduction in Cambodia. The questionnaire was pretested with ten PWID in Phnom Penh, who were later excluded from the main study.

We collected socio-demographic information including age (continuous), gender, type of the community (urban or rural), years of formal education attained (continuous), average income in the past 6 months (continuous), living situation, and employment status. Information on drug use included types of illicit drugs, frequency of use, and needle and syringe exchange practices in the past 3 months. Sexual behaviors included number of partners and condom use with non-commercial and commercial partners (defined as a partner with whom the participant had sex in exchange for money or goods) in the past 3 months. Participants were also asked about community-based HIV and harm reduction services they had received in the past 6 months. Other information included HIV testing, symptoms of sexually transmitted infections (STIs) and care seeking behaviors for the most recent symptoms, use of other substances (tobacco and alcohol) in the past 3 months, as well as experiences with drug rehabilitation and methadone maintenance therapy in the past 12 months.

### Data analyses

All analyses were estimated with sampling weights that corrected for nonresponse and sampling design [26]. The prevalence of HCV antibody was calculated by dividing the total number of HCV antibody positive participants with the total number of participants tested. Socio-demographic characteristics and behavioral variables of HCV antibody positive participants were compared to those of HCV antibody negative participants using Chi-square test (or Fisher’s exact test for an expected cell value of < 5) for categorical variables and Student’s *t* test or Mann-Whitney *U* test for continuous variables. Age, level of education, and income were transformed into categorical variables. To identify independent risk factors for HCV antibody positivity, variables associated with HCV antibody positivity at a significance level of *p* < 0.05 in bivariate analyses were simultaneously included in a multivariable logistic regression model. Because of their epidemiological importance, we included age, gender, level of education, and income in the model regardless of the significance level in bivariate analyses. STATA Version 12.0 for Windows (Stata Corp, TX, USA) was used for the analyses.

### Ethical considerations

This study was approved by the National Ethics Committee for Health Research (NECHR) of the Ministry of Health, Cambodia (No. 193 NECHR). Privacy of the respondents was protected by having the data collected in a private place and confidentiality ensured by removing all personal identifiers from the study documents. Participants were fully informed about the risks and benefits they may expect from their participation and the voluntary nature of the study. A written informed consent was obtained from each participant prior to the data collection.

## Results

### Prevalence of HCV

This study included 286 PWID with a mean age of 31.6 (SD = 7.5). The majority of PWID in the survey (72.0%) were residing in Phnom Penh. Of the total, 30.4% were tested positive for HCV antibody (95% CI = 25.3–36.0%). Almost one-third (31.0%) of the HCV antibody positive individuals were co-infected with HIV. All HCV antibody positive cases were found in Phnom Penh. Only 11.5% of the HCV antibody positive cases were aware of their HCV infection status prior to the study, and five had received HCV treatment.

### Hepatitis C-related characteristics

As shown in Table 1, 21.3% of the study participants reported having been tested for HCV and 15.8% receiving HCV education through drop-in centers or peer outreach workers in the past 12 months. Only 20.8% perceived that their HCV risk was higher than that of the general population.

**Table 1:** Hepatitis C-related characteristics among HCV antibody positive and negative PWID

Hepatitis C-related characteristics	Total (*n* = 286)	HCV antibody test result	
		Positive (*n* = 87)	Negative (*n* = 199)
	*n* (%)	*n* (%)	*n* (%)
Tested for HCV in lifetime	61 (21.3)	28 (32.2)	33 (16.6)
Tested for HCV in the past 12 months (*n* = 61)	47 (77.0)	22 (78.6)	25 (75.8)
Diagnosed or told about having hepatitis C (*n* = 61)	16 (26.2)	10 (35.7)	6 (18.2)
Currently on hepatitis C treatment (*n* = 16)	8 (50.0)	5 (50.0)	3 (50.0)
Received education on hepatitis C in the past 12 months	45 (15.8)	17 (19.5)	28 (14.1)
Source of information on hepatitis C received last time			
Peer educator/outreach worker	23 (50.0)	7 (41.2)	16 (55.2)
Drop-in center	27 (58.7)	13 (76.5)	14 (43.3)
Perceived HCV risk compared to the general population			
Higher	59 (20.8)	24 (27.6)	35 (17.8)
About the same	75 (26.4)	21 (24.1)	64 (27.4)
Lower	32 (11.3)	6 (6.9)	26 (13.2)
Do not know	118 (41.5)	36 (41.4)	82 (41.6)

*HCV* hepatitis C virus, *PWID* people who inject drugs

Chi-square (or Fisher’s exact test when a cell count was smaller than 5) was used

### Socio-demographic characteristics

Table 2 shows that 92.0% of the participants were recruited from urban communities; 72.4% were male; and 20.4% were in Vietnamese ethnic group. Less than half (40.2%) were married, and 57.3% had attained primary education or lower. While 39.9% reported living with their family or relatives, 26.9% were living on the streets. The most common job was a laborer or farmer (37.1%), and 12.9% were unemployed. The majority of the participants (78.7%) reported an average monthly income in the past 6 months of < US$200. A significantly higher proportion of HCV antibody positive participants were living in an urban community (100% vs. 88.4%), were in the age group of ≥ 35 (49.4% vs. 33.7%), were in Vietnamese ethnic group (46.0% vs. 9.1%), had attained primary education or lower (70.1% vs. 51.8%), and lived on the streets (54.0% vs. 15.1%) compared to that in HCV antibody negative group.

**Table 2:** Socio-demographic characteristics of HCV antibody positive and negative PWID

Socio-demographic characteristics	Total (*n* = 286)	HCV antibody test result		
		Positive (*n* = 87)	Negative (*n* = 199)	
	*n* (%)	*n* (%)	*n* (%)	*P* value^a^
Living in an urban community	263 (92.0)	87 (100.0)	176 (88.4)	< 0.001
Male gender	207 (72.4)	66 (75.9)	141 (70.9)	0.38
Age group				< 0.001
18–24	54 (18.9)	1 (1.1)	53 (26.6)	
25–34	122 (42.7)	43 (49.4)	79 (39.7)	
≥ 35	110 (38.5)	43 (49.4)	67 (33.7)	
Ethnic group				< 0.001
Khmer	227 (79.6)	47 (54.0)	180 (90.9)	
Vietnamese	58 (20.4)	40 (46.0)	18 (9.1)	
Current marital status				0.44
Never married	114 (39.9)	30 (34.5)	84 (42.2)	
Married	115 (40.2)	37 (42.5)	78 (39.2)	
Widowed/divorced/separated	57 (19.9)	20 (23.0)	37 (18.6)	
Level of formal education completed				0.009
Primary (0–6 years)	164 (57.3)	61 (70.1)	103 (51.8)	
Secondary school (7–9 years)	68 (23.8)	17 (19.5)	51 (25.6)	
High school or higher (≥ 10 years)	54 (18.9)	9 (10.3)	45 (22.6)	
Living arrangement				< 0.001
On the street (homeless)	77 (26.9)	47 (54.0)	30 (15.1)	
With family or relatives at home	114 (39.9)	21 (24.1)	93 (46.7)	
In own dwelling	52 (15.0)	8 (9.2)	44 (22.1)	
With friends	15 (5.2)	6 (6.9)	9 (4.5)	
Other	28 (9.8)	5 (5.7)	23 (11.6)	
Main occupation				0.41
Unemployed	37 (12.9)	11 (12.6)	26 (13.1)	
Entertainment worker	30 (10.5)	7 (8.0)	23 (11.6)	
Office worker	75 (26.2)	29 (33.3)	46 (23.1)	
Laborer/farmer	106 (37.1)	28 (32.2)	78 (39.2)	
Other	38 (13.3)	12 (13.8)	26 (13.1)	
Average monthly income in the past 6 months (US$)				0.53
< 100	105 (36.7)	33 (37.9)	72 (36.2)	
100–199	120 (42.0)	39 (44.8)	81 (40.7)	
≥ 200	61 (21.3)	15 (17.2)	46 (23.1)	

*HCV* hepatitis C virus, *PWID* people who inject drugs

^a^Chi-square (or Fisher’s exact test when a cell count was smaller than 5) was used

### Characteristics of substance use

As shown in Table 3, the median time since they started injecting drugs was 49 months (IQR = 16–120). About half (50.3%) reported “injection” as the method of their first drug use and that they were firstly introduced to drugs by their friends or colleagues (80.4%). Heroin was the most commonly used drugs in the past 3 months (61.9%), followed by methamphetamine (23.8%). About two-thirds (66.7%) reported always using new syringes/needles for drug injection, and 21.6% using needles or syringes that had been used by someone else in the past 3 months. Less than half (41.6%) had been sent to a drug rehabilitation center, and 43.7% had received methadone maintenance therapy in the past 12 months. The use of other substances was also common, with 29.4% reported alcohol drinking at least three times per week; of them, 48.5% reported binge drinking (drinking at least five units of alcohol on a typical day) on at least 3 days per week in the past 3 months.

**Table 3:** Characteristics of substance use among HCV antibody positive and negative PWID

Substance use characteristics	Total (*n* = 286)	HCV antibody test result		
		Positive (*n* = 87)	Negative (*n* = 199)	
	*n* (%)	*n* (%)	*n* (%)	*P* value^a^
Median months using drugs (IQR)	49 (16–120)	96 (36–132)	36 (12–120)	< 0.001
Mode of first drug use—injecting	104 (50.3)	51 (58.6)	93 (46.7)	0.06
Injecting drugs in the past 3 months	250 (88.0)	84 (96.6)	166 (84.3)	0.003
Type of drugs most commonly used in the past 3 months				
Heroin	156 (61.9)	67 (79.8)	89 (53.0)	< 0.001
Yama/ice (methamphetamine)	60 (23.8)	21 (25.0)	39 (23.2)	0.75
Ecstasy	9 (3.6)	0 (0.0)	9 (5.4)	0.27
Always used new syringes/needles in the past 3 months	112 (66.7)	27 (61.4)	85 (68.5)	0.54
Used needles/syringes used by someone else in the past 3 months	37 (21.6)	8 (18.2)	29 (22.8)	0.31
Having been sent to a drug rehabilitation center in the past 12 months	119 (41.6)	55 (63.2)	64 (32.2)	< 0.001
Having received methadone maintenance therapy in the past 12 months	125 (43.7)	68 (78.2)	57 (28.6)	< 0.001
Alcohol drinking ≥ 3 times per week	84 (29.4)	13 (14.9)	71 (35.7)	< 0.001
Binge drinking ≥ 3times per week (*n* = 171)	83 (48.5)	13 (36.1)	70 (51.9)	0.09

*HCV* hepatitis C virus, *IQR* interquartile range, *PWID* people who inject drugs

^a^Chi-square (or Fisher’s exact test when a cell count was smaller than 5) was used for categorical variables and Mann-Whitney *U* test for continuous variables

The median time of using drugs was significantly longer in HCV antibody positive group compared to that in HCV antibody negative group (96 months vs. 36 months). The proportion of participants who reported injecting any kind of drugs (96.6% vs. 84.3%) and injecting heroin (79.8% vs. 53.0%) in the past 3 months was higher in HCV antibody positive group than in HCV antibody negative group. The proportion of participants who reported having been sent to a drug rehabilitation (63.3% vs. 33.2%) and having received methadone maintenance therapy (78.2% vs. 28.6%) in the past 12 months was significantly higher in HCV antibody positive group. A significantly lower proportion of HCV antibody positive participants reported alcohol drinking ≥ 3 times per week in the past 3 months (14.9% vs. 35.7%).

### Sexual behaviors

Table 4 shows that 95.1% of participants reported being sexually active in the past 3 months, with a median number of sex partners of 1.0 (IQR = 0.0–2.0). Only 16.6% reported always using condoms, and 45.4% reported having sex when a partner was intoxicated in the past 3 months. Of those who reported having sex with partners not in exchange for money or gifts (*n* = 108), 8.3% reported always using condoms with the non-commercial partners in the past 3 months. Of the total respondents, 23.3% reported having sex in exchange for money or goods in the past 3 months, of whom 26.8% reported always using condoms with the commercial partners in the past 3 months. Despite their substantial HIV risk, only 27.3% perceived that their HIV risk was higher compared to that of the general population. No significant difference was found in comparison of sexual behaviors in HCV antibody positive and negative group (Table 4).

**Table 4:** Sexual behaviors among HCV antibody positive and negative PWID

Sexual behaviors in the past 3 months	Total (*n* = 286)	HCV antibody test result		
		Positive (*n* = 87)	Negative (*n* = 199)	
	*n* (%)	*n* (%)	*n* (%)	*P* value^a^
Had sexual intercourse	272 (95.1)	84 (96.6)	188 (94.5)	0.56
Median number of sex partners (IQR)	1.0 (0.0–2.0)	1.0 (0.0–1.0)	1.0 (0.0–2.0)	0.48
Always used condom with any partner	29 (16.6)	6 (13.3)	23 (17.7)	0.50
Had sex when a partner was intoxicated	79 (45.4)	23 (51.1)	56 (43.4)	0.37
Had sex with partners not in exchange for money or gift	108 (61.7)	26 (57.8)	82 (63.1)	0.53
Always used condom with partners not in exchange for money or gift	9 (8.3)	2 (7.7)	7 (8.5)	0.89
Had sex in exchange for money or goods	41 (23.3)	6 (13.3)	35 (26.7)	0.07
Always used condom with partners in exchange for money or goods	11 (26.8)	1 (16.7)	10 (28.6)	0.48
Perceived HIV risk compared to the general population				0.48
Higher	78 (27.3)	28 (32.2)	50 (25.1)	
About the same	96 (33.6)	28 (32.2)	68 (34.2)	
Lower	33 (11.5)	7 (8.0)	26 (13.1)	
Do not know	79 (27.6)	24 (27.6)	55 (27.6)	
Perceived HIV risk compared to the general population				
Higher	59 (20.8)	24 (27.6)	35 (17.8)	
About the same	75 (26.4)	21 (24.1)	64 (27.4)	
Lower	32 (11.3)	6 (6.9)	26 (13.2)	
Do not know	118 (41.5)	36 (41.4)	82 (41.6)	

*HCV* hepatitis C virus, *HIV* human immunodeficiency virus, *IQR* interquartile range

^a^Chi-square (or Fisher’s exact test when a cell count was smaller than 5) was used for categorical variables and Mann-Whitney U test for continuous variables

### Access to community-based HIV services

Table 5 shows that 68.9% of the study participants reported having received some form of community-based HIV services in the past 6 months. The services included condom and lubricant distribution (71.5%), HIV/syphilis testing (63.1%), HIV education (48.8%), needle and syringe distribution (50.8%), methadone maintenance therapy (43.7%), drop-in services (22.3%), and legal support (5.4%). The proportion of participants who reported having received needles and syringes from community-based harm reduction programs (68.2% vs. 41.9%) and methadone maintenance therapy (78.2% vs. 28.6%) in the past 6 months was significantly higher in HCV antibody positive group than in HCV antibody negative group.

**Table 5:** Access to community-based HIV and harm reduction services among HCV antibody positive and negative PWID

Access to community-based services in the past 6 months	Total (*n* = 286)	HCV antibody test result		
		Positive (*n* = 87)	Negative (*n* = 199)	
	*n* (%)	*n* (%)	*n* (%)	*P* value^a^
Received community-based services	197 (68.9)	66 (75.9)	131 (65.8)	0.09
HIV education	63 (48.8)	18 (40.9)	45 (52.9)	0.20
Condom and lubricant distribution	93 (71.5)	33 (75.0)	60 (69.8)	0.53
Needle and syringe distribution	66 (50.8)	30 (68.2)	36 (41.9)	0.005
HIV/syphilis testing	82 (63.1)	30 (68.2)	52 (60.5)	0.39
Legal support	7 (5.4)	2 (4.5)	5 (5.8)	0.76
Drop-in services	29 (22.3)	6 (13.6)	23 (26.7)	0.09
Methadone maintenance therapy	125 (43.7)	68 (78.2)	57 (28.6)	< 0.001

*HCV* hepatitis C virus, *HIV* human immunodeficiency virus, *PWID* people who inject drugs, *STI* sexually transmitted infections

^a^Chi-square (or Fisher’s exact test when a cell count was smaller than 5) was used

### Factors associated with HCV antibody

Factors associated with HCV antibody positivity among PWID in this study are shown in Table 6. After adjustment for other covariates, the odds of HCV antibody positivity was significantly higher among participants who were in the older age group of 25 to 34 (AOR = 1.85, 95% CI = 1.06–7.92) and ≥ 35 (AOR = 2.67, 95% CI = 1.24–5.71), were in Vietnamese ethnic group (AOR = 5.44, 95% CI = 2.25–13.14), were living on the streets (AOR = 3.01, 95% CI = 1.29–704), had been sent to a drug rehabilitation center in the past 12 months (AOR = 2.67, 95% CI = 1.21–5.90), had received methadone maintenance therapy in the past 12 months (AOR = 3.02, 95% CI = 1.32–6.92), and were tested positive for HIV (AOR = 3.80, 95% CI = 1.58–9.12) compared to their respective reference group.

**Table 6:** Factors associated with HCV antibody positivity among PWID in multivariate logistic regression model

Variables in the final model	AOR (95% CI)	*P* value
Age group		
< 25	Reference	
25–34	1.85 (1.06–7.92)	0.009
≥ 35	2.67 (1.24–5.71)	0.001
Ethnic group		
Khmer	Reference	
Vietnamese	5.44 (2.25–13.14)	< 0.001
Living arrangement		
With family or relatives	Reference	
In the street (homeless)	3.01 (1.29–7.04)	0.01
In own dwelling	0.64 (0.20–2.03)	0.45
With friends	0.15 (0.46–10.07)	0.33
Having been sent to a drug rehabilitation center in the past 12 months		
No	Reference	
Yes	2.67 (1.21–5.90)	0.02
Having received methadone maintenance therapy in the past 12 months		
No	Reference	
Yes	3.02 (1.32–6.92)	0.009
HIV testing result		
Negative	Reference	
Positive	3.80 (1.58–9.12)	< 0.001

*AOR* adjusted odds ratio, *CI* confidence interval, *HCV* hepatitis C virus, *HIV* human immunodeficiency virus, *PWID* people who inject drugs

Age, gender, marital status, education level, income, and variables associated with HIV infection in the bivariate analyses at a level of *p* < 0.05 were simultaneously included in the model

## Discussion

Findings from this national survey show that the overall prevalence of HCV antibody among PWID in Cambodia was 30.4%. Prior to this study, a few smaller studies in the country had reported varying HCV antibody prevalence in the Cambodian general adult populations, ranging from 2.3 to 14.7% [27–30]. Among Cambodian people living with HIV, previous studies reported a prevalence between 5.3 and 10.5% [11–13]. Although one of these studies included some PWID, the number was small [11].

Our study is the first exclusively conducted among PWID and shows very high prevalence of HCV antibody in this population. However, in this study, confirmation of active infection and genotyping data were not feasible due to cost of HCV ribonucleic acid (RNA) testing, and it was not part of the national IBBS protocol. The prevalence of active hepatitis C would be expected to be lower than the proportion of people with HCV antibody [11]. However, the prevalence of HCV antibody still provides important data related to the potential magnitude of HCV infection and has implications for education, services, and policy in Cambodia. For instance, of the 87 participants who tested positive for HCV, only ten (11.5%) were aware of their status prior to the study, of which only five had received HCV treatment.

Multivariate results showed several factors associated with positive HCV antibody screening results. After adjustment for potential confounding factors, the odds of testing positive for HCV antibody was significantly higher among PWID who were in the older age group of 25 to 34 and ≥ 35, compared with those aged ≤ 25. The association of older age with higher odds of testing positive for HCV antibody is similar to findings in previous studies ([31–34]. It is plausible that, as PWID continue to inject, they have more opportunities to acquire HCV, and indeed, duration of injection has been reported as independently predictive of HCV antibody positivity [31, 32, 35] or seroconversion [36].

Our study suggests that HCV antibody positivity was three times more likely to be detected among participants who were living on the streets compared to those living with their family or relatives at home. Other studies have shown that homelessness is predictive of both the likelihood to inject [37] and to using shared equipment [38]. On a practical level, this finding provides a useful way in which to categorize profiles of HCV risk among PWID, who were also identified as being at higher risk of HIV in this national survey (Mburu M, Chhoun P, Navy Chann, Tuot S, Mun P, and Yi S: Prevalence and risk factors for HIV infection among people who injct drugs in Cambodia: findings from a national survey, submitted). As such, outreach services to homeless PWID will be essential for prevention of both HCV and HIV. This is particularly relevant given that 26.9% of the participants were living on the streets. In addition, the prevalence of HCV antibody was five times higher among participants of Vietnamese ethnic origin. Although ethnicity has been reported to be associated with HCV in other studies [39], we suggest that this is likely to be a proxy indicator of other structural risk behavioral or other risk factors, which would need to be further investigated in our context.

In Cambodia, the national response toward people who use and inject drugs has previously included sending users to a compulsory drug rehabilitation center, which have generally been associated with poor health outcomes [40, 41]. In China, a recent study also found that having been sent to a compulsory drug rehabilitation center increased the odds of positive HCV antibody [33]. While these findings do not necessarily point to causation, they are generally consistent with assertions that structural factors related to arrests and incarceration could promote risky injection and may lead to HCV infection [31]. Indeed, studies from other countries such as Iran, the United Kingdom, and Australia have shown that the odds of positive HCV infection are higher among PWID with history of incarceration [35, 42, 43].

The association between positive HCV antibody and methadone maintenance therapy is not consistent across the literature. Although it was associated with the increased odds of testing positive for HCV antibody in our own and other studies [33, 35], this association may not necessarily be causal. Indeed, several studies indicate that opioid substitution treatment (OST) could be protective of HCV [44, 45]. However, OST could be an indication of longer-term drug use and dependency that may lead to increased HCV risk.

Our observation that being infected with HIV tripled the odds of being positive for HCV antibody is not necessarily surprising, given the most common shared risk factor for both infections—unsafe injection. In our sample, 21.6% reported using needles or syringes that had been used by someone else in the past 3 months. Indeed, the endemic level of HCV among the general population in Cambodia could be related to the historical popularity—and to some extent, unsafe injecting practices in the country [46, 47]. Other studies, such as from Canada and India, have also reported associations between HIV and HCV [48, 49].

### Policy implications

These results provide critical information for programming and policy. First, there is a need to raise awareness of hepatitis C, so as to ensure that detection is increased. Increasing awareness of HCV can also benefit HIV testing, prevention, and treatment [49]. Second, there is a need for increased governmental focus on hepatitis C, with intent of increasing screening, diagnosis, and treatment. Currently, access to pegylated interferon and ribavirin is limited in Cambodia [11]. Poor availability of treatment is known to be associated with poor health outcomes among those with hepatitis C in many low- and middle-income countries [50]. As opposed to treatment with pegylated interferon and ribavirin, however, the advent of new generation direct-acting antiviral (DAA) that are effective across different genotypes [51, 52] could provide impetus for enhancing screening, diagnosis, and treatment. Although, currently, there is no national program to target hepatitis C in Cambodia, MSF has provided hepatitis C screening and treatment using DAAs free of charge since 2016. Given the high prevalence of HCV in PWID, this high-risk population should be the target for both prevention and treatment programs in Cambodia. In this study, using the respondent-driven sampling method, we could reach 87 PWID living with HCV; 88.5% of them who were not aware of their HCV antibody status prior to the study would benefit from the close follow-up and linkage to care and treatment services provided by MSF.

### Limitations of the study

In interpreting our findings, it should be borne in mind that the design, recruitment, methods of data collection, and sample profile could affect what are reported. However, the study aimed to be representative as far as possible by recruiting participants nationally. The cross-sectional nature of this study only captures a snapshot and may not provide valuable information regarding spontaneous clearance of HCV, which is part of natural history of the infection [53]. Personal sensitive data were collected from the participants through a self-reported questionnaire, which can be affected by social desirability bias as is the case in other studies of PWID [54]. In addition, the HCV antibody prevalence data presented in this paper were based on a rapid test, and did not represent current active HCV infection [55]. A recent review of rapid diagnostics reported that OraQuick ADVANCE® had higher sensitivity (98%, 95% CI 97–98%) compared to the other oral brands (pooled sensitivity: 88%, 95% CI 84–92%) [56]. Nevertheless, other diagnostic methods such as polymerase chain reaction (PCR)-based positivity are required to identify active hepatitis C, but was not feasible in this large national survey. Despite this limitation, the oral rapid screening could be a useful strategy for screening PWID who are unaware of their HCV status [57], in keeping with the global testing and elimination targets [55]. This is particularly relevant in Cambodia, given the high prevalence of HCV antibody found in this study.

## Conclusions

Hepatitis C is becoming an important health promotion focus for PWID globally [58]. Understanding the epidemiology of HCV among this population, who are already heavily burdened by HIV, will inform future prevention, care, and treatment of HCV or HCV/HIV co-infection. In Cambodia, HCV/HIV co-epidemic is poorly characterized [58], yet this has relevance for PWID. This article presents findings from a national survey in Cambodian PWID, showing the HCV antibody prevalence of 30.4%. In addition, several important factors including older age, homelessness, Vietnamese ethnicity, having been sent to a drug rehabilitation center, having received methadone maintenance therapy, and being infected with HIV were independently predictive of HCV infection. Given the health impact of hepatitis C, these findings suggest a number of policy and programmatic implications to promote health of PWID who are vulnerable to or infected with HCV in resource-limited settings.

## Abbreviations

CI: Confidence interval
HCV: Hepatitis C virus
HIV: Human immunodeficiency virus
IBBS: Biological and behavioral survey
IQR: Interquartile range
MSF: Médecins Sans Frontières
NECHR: National Ethics Committee for Health Research
NGO: Non-governmental organization
OST: Opioid substitution treatment
PCR: Polymerase chain reaction
PIN: Personal identification number
PWID: People who inject drugs
PWUD: People who use drugs
RDS: Respondent-driven sampling
RNA: Ribonucleic acid
SD: Standard deviation
